# Hemi-Diaphragm Plication and/or Tracheostomy Are Valuable Adjunctive Procedures After Repair of Congenital Heart Defects in Children: A Systematic Review

**DOI:** 10.7759/cureus.48648

**Published:** 2023-11-11

**Authors:** Vishal V Bhende, Tanishq S Sharma, Mathangi Krishnakumar, Amit Kumar, Gurpreet Panesar, Kunal A Soni, Kartik B Dhami, Mamta R Patel, Ashwin S Sharma, Sohilkhan R Pathan, Hardil P Majmudar

**Affiliations:** 1 Pediatric Cardiac Surgery, Bhanubhai and Madhuben Patel Cardiac Centre, Shree Krishna Hospital, Bhaikaka University, Karamsad, IND; 2 Community Medicine, SAL Institute of Medical Sciences, Ahmedabad, IND; 3 Anaesthesiology, St. John's Medical College Hospital, Bengaluru, IND; 4 Pediatric Cardiac Intensive Care, Bhanubhai and Madhuben Patel Cardiac Centre, Shree Krishna Hospital, Bhaikaka University, Karamsad, IND; 5 Cardiac Anaesthesiology, Bhanubhai and Madhuben Patel Cardiac Centre, Shree Krishna Hospital, Bhaikaka University, Karamsad, IND; 6 Central Research Services, Bhaikaka University, Karamsad, IND; 7 Internal Medicine, Gujarat Cancer Society Medical College, Hospital and Research Centre, Ahmedabad, IND; 8 Clinical Research Services, Bhanubhai and Madhuben Patel Cardiac Centre, Shree Krishna Hospital, Bhaikaka University, Karamsad, IND

**Keywords:** tracheostomy, single-ventricle physiology, respiratory failure, pediatric critical care, pediatrics, phrenic nerve, diaphragm plication, diaphragm palsy, congenital heart disease(chd)

## Abstract

Diaphragmatic paralysis (DP), whether unilateral or bilateral, often leads to extended recovery and more severe complications, particularly in neonates and infants undergoing congenital heart surgery. This condition's impact is most pronounced after single-ventricle palliative procedures. Tracheostomy prevalence is rising in pediatric patients with congenital heart disease (CHD) despite its association with high resource utilization and in-hospital mortality. This study examines the reported incidence of diaphragmatic paralysis and timing of tracheostomy in pediatric patients undergoing surgery for congenital heart disease in the literature and a retrospective analysis of cases in our institution between 2018 and 2023, offering insights for prospective management. An electronic search of PubMed databases retrieved 10 studies on pediatric tracheostomy and 11 studies on DP. Our retrospective analysis included 15 patients, of whom 10 underwent tracheostomy, four underwent diaphragmatic plication, and one underwent both.

Postoperative tracheostomy had an 11.8% mortality rate in our systematic review, rising to 40% in our observational study. Diaphragm repair and early diagnosis can reduce morbidity, prevent complications, and improve patients' quality of life.

## Introduction and background

**Figure 1:**
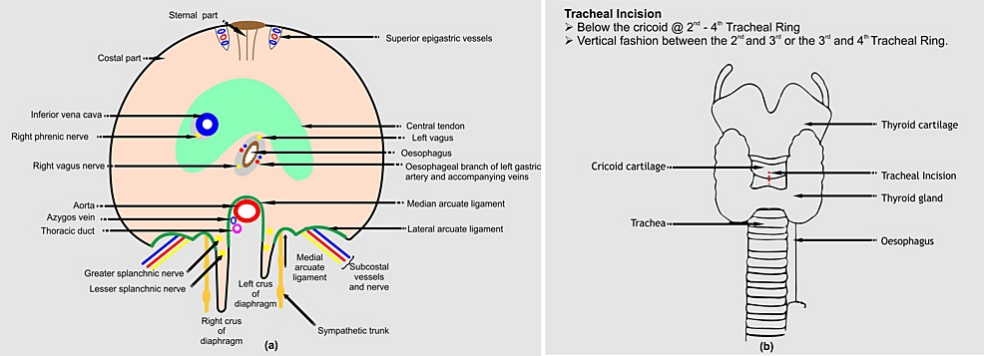
Surgical anatomy of (a) the thoraco-abdominal diaphragm and (b) trachea (Image Credits: Dr. Vishal V. Bhende)

The thoracic and abdominal cavities are divided by the muscular-fibrous diaphragm. It consists of a central fibrous body, upper lumbar vertebrae, and peripheral muscular elements coming from the chest wall. This structure plays a crucial role in respiration and is a primary muscle of inspiration. Innervation is provided by the phrenic nerve, which can sustain damage during diverse cardiac surgical interventions, resulting in the occurrence of unilateral or bilateral diaphragmatic paralysis (Figure 1).

Diaphragmatic paralysis (DP) stemming from phrenic nerve damage, whether reversible or irreversible, is a significant postoperative complication of pediatric cardiac surgery [1,2]. Young patients and those with univentricular physiology commonly present with symptoms of DP, as it potentially disrupts the systemic and pulmonary blood flow dynamics. These include dyspnea, tachypnea, difficulties with extubation, recurrent need for intubation, prolonged ventilation, susceptibility to pneumonia, and an increased risk of sepsis [2]. Timely identification of DP is crucial to improving patient outcomes, necessitating prompt weaning, clinical expertise, and vigilant suspicion.

Typically, the identification of DP is prompted by clinical indications and verified through fluoroscopic assessment of the diaphragm (Table 1).

**Table 1:** Diagnosis of diaphragm palsy DP: diaphragmatic palsy

Sr. No.	Description
01	Paradoxical respiration, atelectasis that doesn’t resolve, weaning failure
02	Hoover's Sign: Infants with DP frequently have indrawing of the lower chest during inspiration.
03	The Kienbock sign: chest radiograph shows an increase in elevation of the hemidiaphragm. This can also be demonstrated with bedside ultrasonography or fluoroscopy when the patient is spontaneously breathing. [1]
04	DP can be recognized on 2D echocardiography as a paradoxical diaphragmatic movement during breathing. Sensitivity ~ 100 %, specificity ~ 81 %.
05	Sniff Test: While sniffing, the affected diaphragm migrates paradoxically higher due to negative intrathoracic pressure. This is seen fluoroscopically. Sensitivity ~ 100 %, specificity ~ 74 %

The recuperation of diaphragmatic function spans a variable period, ranging from days to months, with the possibility of incomplete recovery, underscoring an unpredictable trajectory [3]. However, relying on the natural recovery of diaphragmatic function results in extended periods of mechanical ventilation. Conversely, early diaphragmatic plication is beneficial, resulting in swift extubation, reduced durations of intensive care unit (ICU) and hospital stays, minimized morbidity, and improved outcomes [4,5]. Nevertheless, the timing of this plication procedure varies, encompassing both early and late interventions (Table 2).

**Table 2:** Children with DP who underwent open heart surgery and indications for a diaphragmatic plication

Sr. No.	Description
01	Age under 6 months
02	Respiratory distress
03	Tachypnea
04	Oxygen dependency
05	CO_2_ retention
06	Unable to disconnect from the ventilator
07	Children who have cavopulmonary shunts to stop the rise in pulmonary vascular resistance

The survival rates among pediatric CHD patients have improved with advancements in surgical methodologies that address even the most intricate structural anomalies and enhanced capabilities within ICUs. However, while more children with complex CHD and co-morbidities achieved long-term survival, some still require tracheostomy for airway safeguarding and/or dependence on ventilatory support. Many undergo prolonged and challenging recovery and post-cardiac procedures, and even after the initial recovery phase, children with CHD exhibit heightened susceptibility to subsequent critical ailments.

The objectives of this study were twofold: (i) to conduct a thorough analysis of our data from a retrospective study of pediatric cardiac surgery patients, as well as the literature that already exists on DP, and (ii) to perform a systematic review in order to assess tracheostomy's indications, performance, outcomes, and resource utilization in children with CHD.

## Review

Method

Study Objective

The preferred reporting of systematic reviews and meta-analyses (PRISMA) standards were followed during the preparation of the systematic review to guarantee transparent and thorough reporting of the study methodology and conclusions.

From our internet database (FTP 192.168.0.5), information about 15 patients was retrieved. We enlisted patients who received tracheostomy or diaphragm plication surgeries between 2018 and 2023 at the Bhanubhai and Madhuben Patel Cardiac Centre, Karamsad, Anand, Gujarat, India.

Search Strategy 

Diaphragm Plication: A systematic literature search was conducted. Keywords used to search for relevant articles included “diaphragm”, “plication”, “paresis”, “pediatric”, “infants”, “neonates”, “cardiac surgery”, and “congenital heart disease” from 1978 until 2012, PubMed was searched using the Boolean logical operations AND and OR (Diaphragm Plication). This study period of search was used to bring out the early practice of surgery and its impact on diaphragm palsy. Only studies with a minimum of 500 patients were taken. Case reports, expert opinions, and conference abstracts were not considered in the study. The search was restricted to articles published in the English language.

Tracheostomy Mortality: The following keywords were used in the PubMed database: “paediatric tracheostomy mortality infant”. The search was restricted to paediatric age group (0-10 years) and in the English language. Only original research articles, cohorts, case series, and systematic reviews were considered in the time period from January 2016 to June 2020. Articles not mentioning tracheostomy mortality were excluded. This time period was chosen to capture the data exactly during our study period for comparability. Due to the heterogenicity of the data, doing a meta-analysis was not feasible.

Data Extraction

To gather pertinent information from the included research, a consistent data extraction form was created. The current study is a retrospective observational study, and the Institutional Ethics Committee (IEC-2) at Bhaikaka University in Anand, Gujarat, India, approved the study protocol on September 9, 2023, vide Approval No. IEC/BU/2023/Cr. 34/248/2023. Data on 15 patients who underwent tracheostomy and/or diaphragm plication procedures at the Bhanubhai and Madhuben Patel Cardiac Centre, Karamsad, Anand, Gujarat, India, between 2018 and 2023 were taken from our computerized cardiovascular database (FTP 192.168.0.5).

*Reporting* 

**Figure 2:**
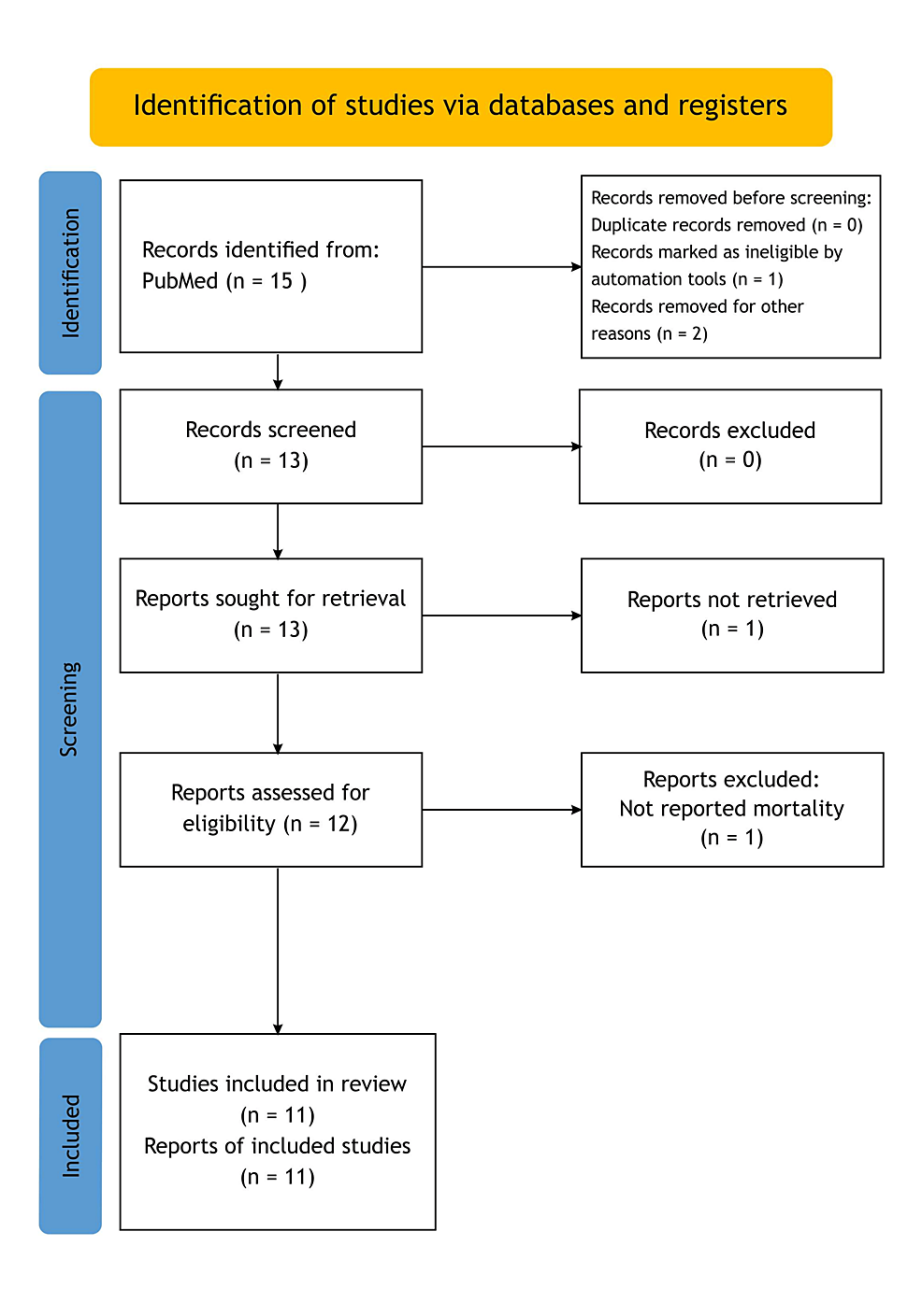
PRISMA 2020 flowchart for new systematic reviews that only involved database and registration searches. *Page MJ, McKenzie JE, Bossuyt PM, Boutron I, Hoffmann TC, Mulrow CD, et al. The PRISMA 2020 statement: an updated guideline for reporting systematic reviews. BMJ 2021;372:n71. doi: 10.1136/bmj.n71 For more information, visit: http://www.prisma-statement.org/

**Figure 3:**
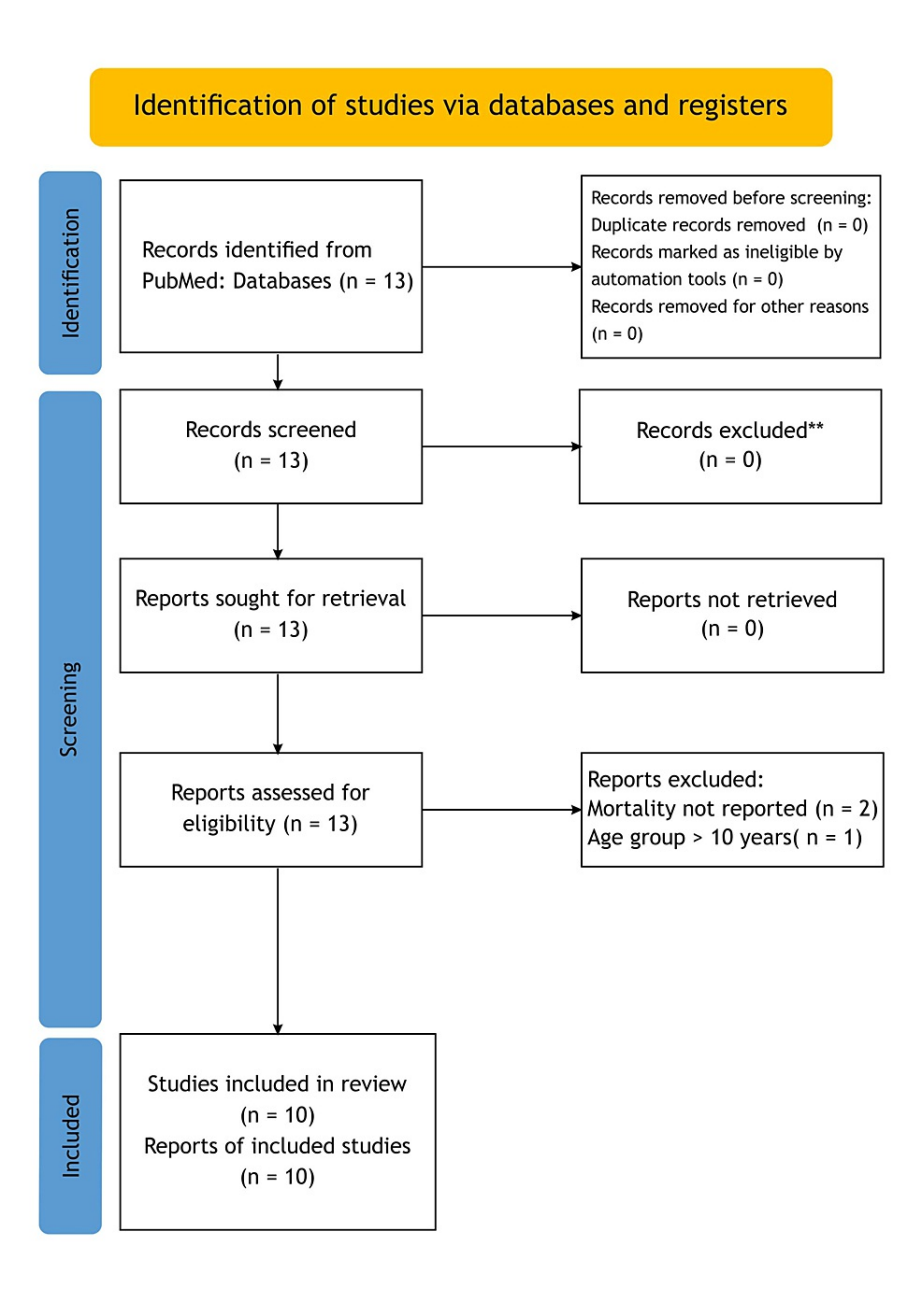
PRISMA search approach for paediatric tracheostomy mortality from 2016 to 2020. **To review the literature for relevant information on paediatric tracheostomy mortality. The following keywords were used: “paediatric tracheostomy mortality”. The search was restricted to the paediatric age group (0-10 years) and in the English language. Only original research articles, cohorts, case series, and systematic reviews were considered in the time period from January 2016 to June 2020. Articles not mentioning tracheostomy mortality were excluded. This was done to capture the data exactly during our study period for comparability. Due to the heterogenicity of the data, doing a meta-analysis was not feasible.

To guarantee transparent and thorough reporting of the study methodology and findings, this systematic review was created in accordance with PRISMA principles (Figures 2, 3).

A comprehensive search of electronic databases yielded 11 articles for the studies on the incidence of DP and 10 articles from the tracheostomy group. Several studies investigating the causes of diaphragmatic paralysis (DP) in pediatric patients have examined its occurrence following heart surgery and have reported a range of incidence rates, typically spanning from 0.28% to 5.64% [6-16]. Notably, DP appears to be more prevalent after specific cardiac procedures, such as ventricular septal defect (VSD) closure, bidirectional Glenn or Fontan operation, surgery for tetralogy of Fallot (TOF), the classic or modified Blalock-Taussig (BT) shunt, systemic to pulmonary artery shunts, and arterial switch operation.

Akay's research [8] highlighted elevated DP rates following BT shunt (11.1%), TOF correction (31.5%), and VSD closure with pulmonary artery patch plasty (11.1%). Furthermore, the incidence of DP necessitating diaphragmatic plication was notably higher after BT shunt (23.8%), TOF correction (11.9%), and arterial switch operation (19%) [8]. Another study by John-Arreola et al. [9] also noted an increased DP occurrence after arterial switch operation, BT shunt, and Fontan.

In addition, Watanabe et al. [15] reported DP incidence rates of 6.7% after the mustard procedure, 5.6% following right ventricular outflow tract reconstruction, and 2.7% following TOF repair. Among patients undergoing closed-heart procedures, DP was observed in 6.2% after the Glenn anastomosis, 5.9% following the Blalock-Hanlon atrial septectomy, and 5.1% following the right BT shunt. An overview of the key findings from these selected studies is presented in Table 3.

**Table 3:** Studies on the incidence of diaphragmatic palsy *data not available; DP: diaphragmatic palsy; n: number

Author/ Year	Duration of study (years)	Incidence (%)	No of Patients (n)	No. with DP	Time to plication (days)
Dagan, 2006 [6]	10	0.28	3214	9	*
Lemmer,2006 [7]	14	1.4	5128	74	*
Akay, 2006 [8]	10	4.9	3071	152	12
Joho Arreola, 2006 [9]	6	5.4	802	43	21
Van Onna , 1998 [10]	5	1.9	867	17	5
Vazquez, 1996 [11]	5	2.3	556	13	*
Tonz, 1996 [12]	-	1.5	1656	25	15-110
Picardo, 1996 [13]	-	0.73	3400	25	*
Serraf, 1989 [14]	10	1.2	9149	109	18
Watanabe, 1986 [15]	12	1.6	7670	125	14
Mickell, 1978 [16]	8	1.7	1891	32	*

Because pediatric tracheostomies are required due to children's increased medical complexity, they are associated with high fatality rates. The mortality rate varies from less than 1% in patients having surgery for obstructive sleep apnea (OSA) to 26% in those with hypoplastic left heart syndrome [17-26]. The chosen studies are summarized in Table 4.

**Table 4:** Post-tracheostomy mortality RC: retrospective cohort; n: number

Primary author	Year	Design	Number of patients (n)	Tracheostomy-related mortality rate (%)	Mortality rate (%)
Watters [17]	2016	RC	502	-	9.0%
Funamura [18]	2016	RC	513	3.5%	16.6%
Tsuboi [19]	2016	RC	212	1.9%	14% , 29%
Dal’Astra [20]	2017*	Meta-analysis	5933	0.9%	10.6%
McPherson [21]	2017	RC	426	-	23%
Rizzi [22]	2017	Case series	29	0%	0%
Prodhan [23]	2017	RC	126	-	26%
Rawal [24]	2019	RC	543	-	4.3%
Han [25]	2020	Prospective cohort	3442	-	18.5%
Friesen [26]	2020	RC	14,155	-	8.6%

Based on the results of the literature review, we have created a master chart of patients enrolled in our study. The master chart is depicted in Table 5.

**Table 5:** Masterchart of patients enrolled in the study RV: right ventricle; SVC: superior vena cava; VSD: ventricular septal defect; ASD: atrial septal defect; PDA: patent ductus arteriosus; AV: atrio-ventricular; AVVR: atrio-ventricular valve regurgitation; LV: left ventricle; DORV: double outlet right ventricle; PA: pulmonary artery; PAH: pulmonary arterial hypertension; CCHD: complex cyanotic congenital heart disease; TCA: total circulatory arrest; ICU: intensive care unit; POD: post-operative day; R/L: right/left; P: patient; M/F: male/female

Patient ID	Age in Months	Sex M/F	Height cms.	Weight kgs.	Diagnosis	Surgical procedure POD O	Diaphragm Plication R/L	Time to Plication (Days)	Time to Extubation (Days/Hours)	Ventilator (Days/Hours)	Hospital Stay (Days)	Length of ICU stay (Days)	Tracheostomy performed (Days)	Tracheostomy tube Cuffed/ Non-cuffed	Tracheostomy Tube size
P 1	6	M	63	5.6	Tricuspid Atresia Type IB, Large Atrial Septal Defect, Restricted Ventricular Septal Defect, Pulmonary Stenosis, RV Hypoplastic, Bilateral SVCs (Univentricular Physiology)	Pulsatile Bilateral Bi-directional Glenn shunt + Partial MPA ligation/Tightening Off-pump	Left	POD 18	52 / 1248	52 / 1248	59	54	POD 41	Cuffed (Rusch)	4 Fr.
P 2	6	F	63	4.5	Ventricular Septal Defect, Atrial Septal Defect, Patent Ductus Arteriosus, Severe Pulmonary Arterial Hypertension (PAH), Down’s Syndrome, Hypothyroidism.	VSD Closure + ASD Closure + PDA Ligation	-	-	30 / 720	30 / 720	31	22	POD 15	Non-cuffed (HANSRAJ)	4 Fr.
P 3	9	M	63	6.5	Common Complete Balanced AV Canal Defect, Rastelli Type A, Moderate AVVR, PDA, Severe PAH	Two patch technique repair of AV canal defect + PDA ligation	-	-	68 / 1632	68 / 1632	82	71	POD 11	Cuffed (Rusch)	4 Fr.
P 4	6	F	51	2.6	Complex Cyanotic Congenital Heart Disease (CCHD), Mitral Valve Atresia, Restrictive Atrial Septal Defect, Hypoplastic LV, DORV, Large VSD, PDA, Severe PAH, Univentricular Physiology.	Atrial Septectomy + PA Banding + PDA Ligation	-	-	68 / 1632	68 / 1632	78	68	POD 36	Cuffed (Rusch)	3.5 Fr.
P 5	2	M	54	3	Multiple Ventricular Septal Defect, Atrial Septal Defect, Patent Ductus Arteriosus, Severe PAH	Multiple VSDs Closure + PDA ligation	-	-	25	25	46	27	POD 16	Cuffed (Rusch)	3.5 Fr.
P 6	3	M	57	3.9	DORV, VSD, Severe PAH	Intra-Ventricular Tunnel Repair (IVTR)	-	-	54	54	69	54	POD 41	Cuffed (Rusch)	3.5 Fr.
P 7	5	M	55	3.54	CCHD, dor, Multiple VSDs amounting to Single Ventricle, Bilateral SVCs, Severe PAH	Pulmonary Artery Banding	Left	POD 15	28	28	36	28	-	-	-
P 8	3	M	54	3.35	Hypoplastic Aortic Arch, Ductal Arch [large PDA continuing as descending aorta], DORV, VSD, Severe PAH	Midline Hypoplastic Aortic Arch Repair {Goretex Interposition Graft 5 mm.} + PDA Division and Suturing + PA Banding under TCA	-	-	-	-	Expired	-	POD 24	Cuffed (Rusch)	3.5 Fr.
P 9	4	M	58	3.5	Multiple apical ventricular septal defects, atrial septal defect, moderate to severe PAH	VSD Closure using Custom-made low-profile PTFE single disc device + glutaraldehyde-treated pericardial patch closure of ASD + PA Banding	-	-	-	-	Expired	-	POD 45	Cuffed (Rusch)	3.5 Fr.
P 10	4	F	58	4.42	Multiple VSDs, ASD, PDA, Down’s Syndrome	Multiple VSDs Closure + PDA ligation	Right	POD 11	13	13	23	16	-	-	-
P 11	4	M	60	4.22	Ventricular Septal Defect, Atrial Septal Defect, Patent Ductus Arteriosus, Mild Tricuspid Regurgitation, Severe PAH	VSD Closure + ASD Closure + PDA ligation	Left	POD 7	8 / 192	8 / 192	16	11	-	-	-
P 12	2	M	52	2.420	Double outlet Right Ventricle (DORV) Moderate Atrial Septal Defect, Large Non-routable Ventricular Septal Defect, Mild Hypoplastic tortuous distal part of Aortic Arch, Severe PAH.	Pulmonary Artery Banding	-	-	-	-	Expired	-	POD 34	Non-Cuffed (Blue Line/ Smith’s Medical)	3 Fr.
P 13	6	F	62	4.050	Ventricular Septal Defect, Mild Mitral Regurgitation, Mild Tricuspid Regurgitation, Severe PAH.	VSD Closure	-	-	22 / 549	22 / 549	125	131	POD 24	Non-Cuffed (Blue Line/ Smith’s Medical)	3.5 Fr.
P 14	4	F	57	3.2	Ventricular Septal Defect, Mild Tricuspid Regurgitation, Severe PAH.	VSD Closure	Left	POD 15	8 / 235	8 / 235	29	25	-	-	-
P 15	8	F	61	3.6	Common Complete AV Canal Defect, Rastelli Type A Moderate Atrio-ventricular valve Regurgitation, Bilateral Superior vena cava, Severe PAH.	Two patch Technique Repair of Complete AV Canal Defect	-	-	-	-	Expired	-	POD 40	Non-Cuffed (Steri-med)	3.5 Fr.

**Figure 4:**
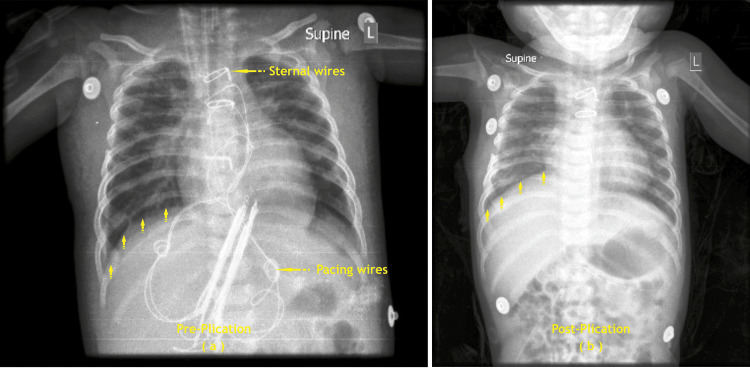
Right diaphragm plication for P 10 patients – (a) Pre-plication, (b) Post-plication P: patient (Image credit: Dr. Vishal V. Bhende)

**Figure 5:**
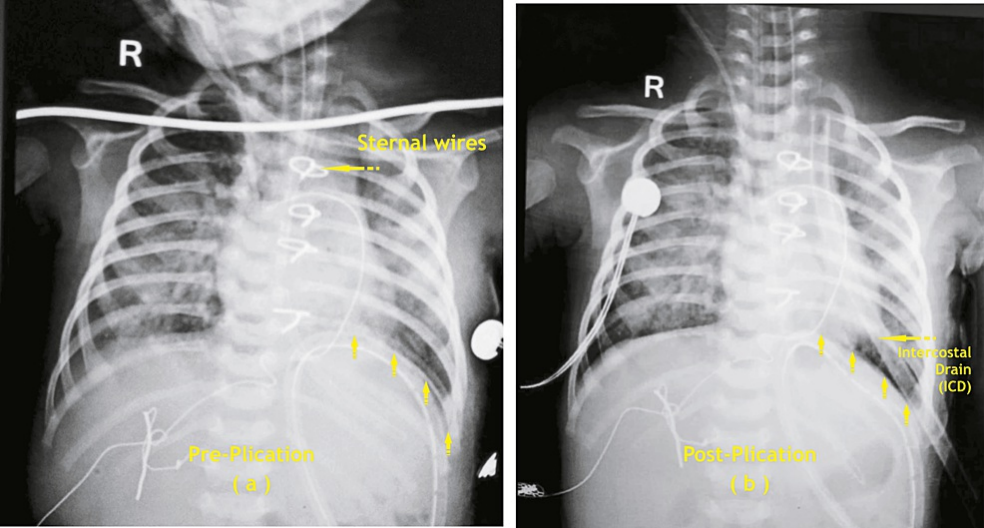
Left diaphragm plication for P 14 patient: (a) Pre-plication, (b) Post-plication P: patient (Image credit: Dr. Vishal V. Bhende)

The pre-plication and post-plication X-ray images of patients 10 and 14 are shown below (Figures 4, 5).

Statistical Analysis

Descriptive statistics are used to represent the baseline characteristics of the study participants, including mean (SD), median interquartile range (IQR), range, and frequency (%). The statistical software STATA 14.2 is used for data analysis.

Results

**Figure 6:**
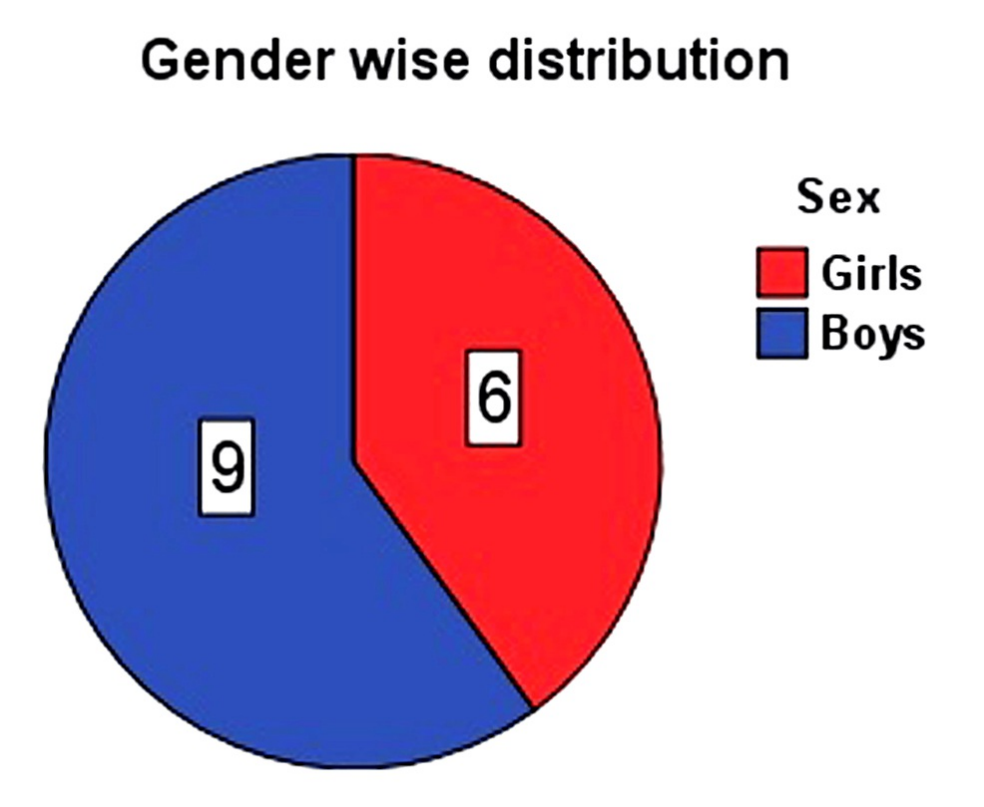
Gender-wise distribution of patients in our study (n = 15)

**Figure 7:**
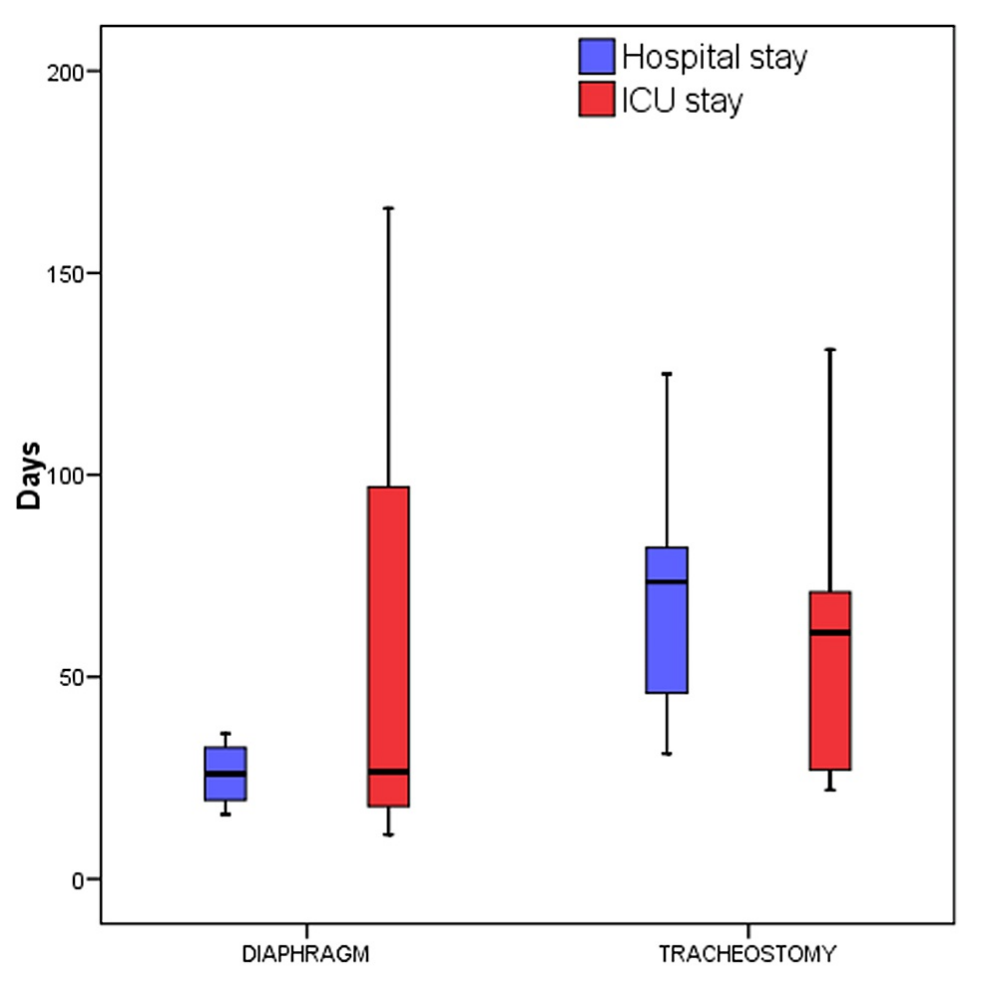
Box plot depicting hospital/ICU stays of the study population for both procedures ICU: Intensive care unit

**Figure 8:**
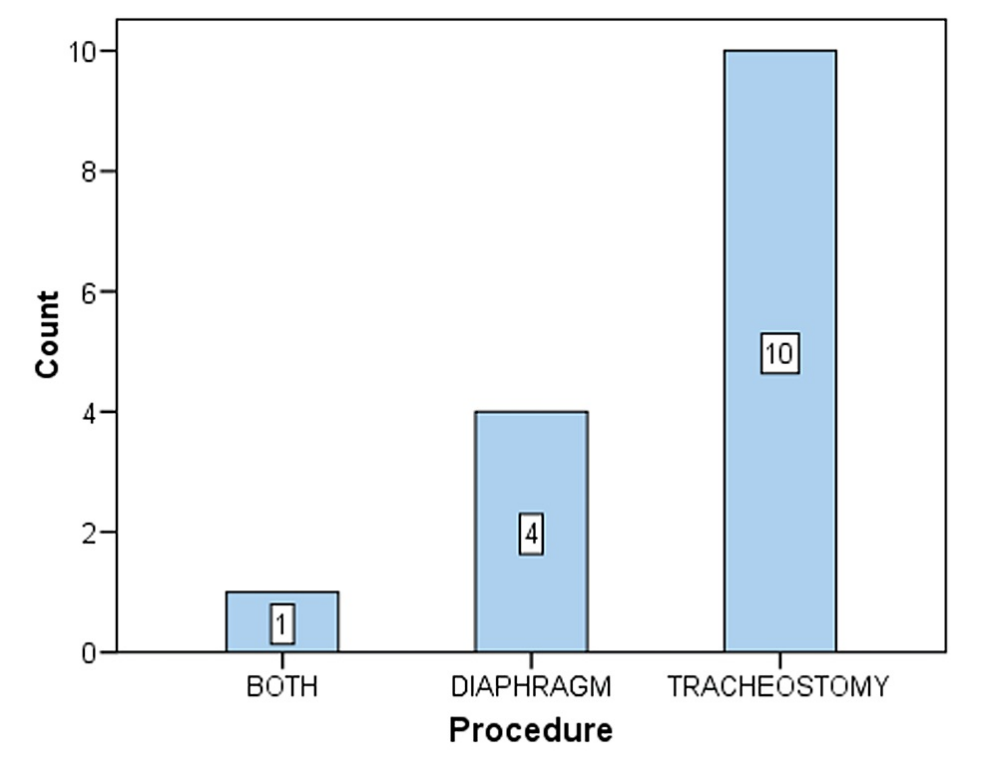
Bar chart depicting both procedures

Of 15 infants, nine were boys, and six were girls, and the mean (SD) age was 4.8 (2) months. The range of age (in months) was 2-9. The mean (SD) height and weight were 57.8 (4.1) cm and 3.8 (1.1) kg, respectively. Tracheostomy and diaphragm plication were performed in 10 and four infants, respectively, and both procedures were followed in one infant (four left-side and one right-side diaphragm). The mean (SD) postoperatively for performing diaphragm plication was 28 (12). Four deaths were reported in the tracheostomy group (n = 10), or approximately 40%. Among them, a cuffed tube was used in two patients, and a non-cuffed tube was used in the other two patients. For infants in whom diaphragm plication was performed, mean (SD) plication days, ventilator days, and hospital stay days were 12(4), 14(9), and 26(9), respectively. The median (IQR) ICU stay days were 27 (18, 97), and the median (IQR) hospital stay was 26 (19, 32). In infants that underwent tracheostomy, the mean (SD) on ventilator days was 44 (21), and in-hospital stay days were 71 (32). The median (IQR) ICU stay days were 61 (27, 71), and the median (IQR) hospital stay was 73 (46, 82) (Figures 6-8).

The outcome of four patients in the tracheostomy group and the causative organisms identified are summarized in Table 6.

**Table 6:** Patients with causative organisms pertaining to infection and cause of death P: patient

Sr. No.	Patient ID	Adjunctive procedure done	Causative organisms identified	Cause of death
01	P 8	Tracheostomy 3.5 Fr. Cuffed	Specimen-Endotracheal secretion (ES) Organism - I Klebsiella pneumoniae isolated after 48 hours of aerobic incubation. Colony Count >10e^5^ cfu/ml	Acute respiratory distress syndrome
02	P 9	Tracheostomy 3.5 Fr. Cuffed	Specimen-ES Organism - I Pseudomonas Aeruginosa (PA) isolated after 48 hours of aerobic incubation. Colony Count >10e^5^ cfu/ml	Cardiogenic Shock
03	P 12	Tracheostomy 3.5 Fr. Non-Cuffed	Specimen- ES Organism - I PA isolated after 48 hours of aerobic incubation. Colony Count >10e^5^ cfu/ml	Sepsis with Pulmonary Haemorrhage
04	P 15	Tracheostomy 3.5 Fr. Non-Cuffed	Specimen- ES	Acute respiratory distress syndrome on chronic respiratory disease.
			First culture Organism - I Klebsiella pneumonia(Enterobacteriaceae) isolated after 48 hours of aerobic incubation. Colony Count >10e^5^ cfu/ml	
			Second culture Organism – I Acinetobacter baumani isolated after 48 hours of aerobic incubation. Colony Count >10e^5^ cfu/ml	
			Third culture Organism - II Burkholderia Cepacia (Non-Enterobacteriaceae) isolated after 48 hours of aerobic incubation. Colony Count >10e^5^ cfu/ml	

Discussion

The reported incidence of DP ranges from 0.33% to 5.7% in the present systematic review analysis [4-7] and from 0.5% to 12.8% in prospective studies [10]. This difference could be related to the asymptomatic nature, uneventful recovery, or diagnostic difficulties associated with DP. In our retrospective observational analysis, after juvenile heart surgery, there were four left-sided and one right-sided case of DP, for a DP incidence of 1.09%. Among our cases, two patients with univentricular physiology necessitated diaphragm plication, while three bi-ventricular physiology patients underwent the procedure. Our inclination toward early plication in univentricular patients, even in cases of compensated respiratory status, may have influenced this pattern. Patients with univentricular physiology have increased respiratory effort and decreased passive venous pulmonary flow due to the absence of negative intrathoracic pressure during inhalation and elevated pulmonary vascular resistance. These result in elevated systemic venous pressure, ascites, and pleural effusion [1,7,27,28]. Diaphragm plication, in such cases, ameliorates pulmonary hemodynamics and reduces systemic venous pressure [29].

A conservative approach comprising 4-6 weeks of extended mechanical ventilation was the gold standard [9,30]. Diaphragm plication has gained widespread acceptance as the conventional treatment for DP cases with reduced cardio-respiratory function. However, the timing of plication differs across studies, ranging from early to late intervention; in the present study, the mean (SD) time to plication was 28 (12) days (four weeks).

Tracheostomy in pediatric patients is associated with higher complication rates compared to adults [17,18]. Pediatric tracheostomy is associated with complications like infection, pneumothorax, obstruction, and fistula [20]. Many studies cited in Table 4 indicated mortality rates ranging from 10% to 20% over at least a year; one reported an 8.6% mortality rate before discharge [26]. However, recent trends indicate reductions in tracheostomy-related mortality rates, which are probably due to international efforts to enhance treatment for these complicated patients [18,20]. Although tracheostomy-specific mortality remains low, it is significant (0.5%-3.5%). In only two recent studies, seven deaths related to tracheostomy were reported [18,19]. The most important risk factors for tracheostomy-related mortality are age associated co-morbidities and duration of stay [26].

In a large cohort, authors demonstrated that the indication of tracheostomy influences mortality. In this study, patients requiring tracheostomies for airway-related complications had the lowest mortality. However, patients requiring treatment for neurological disease had the highest mortality [20]. Compared to children who have the treatment for underlying pulmonary disorders, children who require tracheostomy due to underlying neoplasms or congenital cardiac defects typically have greater mortality rates [17,18,26]. In the present study, four tracheostomy-related deaths occurred among 10 tracheostomized patients. The most common causes were acute respiratory distress syndrome and septic shock. This could be attributed to the longer duration of mechanical ventilation and the underlying condition. Sicker babies are likely to undergo tracheostomies as the duration of mechanical ventilation increases. This could have been the reason for the mortality, not the procedure per se.

With the advancement of techniques and improved intensive care management, complications and mortality have significantly reduced in patients undergoing tracheostomy for prolonged mechanical ventilation [20]. However, the advantage of early plication over tracheostomy is to be determined. Proper patient selection and anticipation of complications will contribute to a better patient outcome in pediatric cardiac surgery.

Limitations

Although this systematic review provides insightful information, it is crucial to highlight some drawbacks, including the limited sample size, the single-center study, and the potential for publication bias to reduce the overall reliability of the results. Nevertheless, this systematic review contributes to the existing knowledge about adjunctive procedures in pediatric cardiac surgery. A systematic review was done, but it was restricted to a few time frames, and a meta-analysis was impossible because of the heterogeneity of the data. The data collected was retrospective in nature. To ascertain the superiority or mortality benefit of plication over tracheostomy was not possible due to the limited sample size. Larger studies are required to answer this question.

## Conclusions

Children who undergo open heart surgery are relatively susceptible to developing DP, which can have serious consequences for morbidity and mortality. Early diagnosis demands a heightened level of suspicion, and management approaches should be tailored to the specific clinical context. For those undergoing univentricular repair, prompt diaphragmatic plication is recommended. Furthermore, this study highlights tracheostomy and its associated resource implications. The observed practice patterns and outcomes indicate that tracheostomy is increasingly performed for congenital heart defects in children. However, it is frequently delayed (by an average of 44 days) when performed concurrently with heart surgery.
